# Parkin Disease and the Lewy Body Conundrum

**DOI:** 10.1002/mds.25486

**Published:** 2013-05-07

**Authors:** Karen M Doherty, John Hardy

**Affiliations:** Reta Lila Weston Institute of Neurological Studies and Research Laboratories, Departments of Molecular Neuroscience and Clinical Neuroscience, University College London Institute of NeurologyLondon, United Kingdom

A clear pathologic hallmark like that identified in sporadic Parkinson's disease (PD) is lacking in many of the monogenic causes of PD. In leucine-rich repeat kinase 2 (*LRRK2*) mutations (PARK8), alpha-synuclein pathology in the form of Lewy bodies (LBs) is frequently, but not consistently, observed. Other pathologies reported in *LRRK2* cases include tau inclusions of the Alzheimer, progressive supranuclear palsy, and frontotemporal lobar degeneration types, and TAR DNA-binding protein 43 (TDP-43) inclusions.^1^ There has been only a single report of a compound heterozygous, phosphatase and tensin homolog (PTEN)-induced, putative kinase 1 (*PINK1*; PARK6) patient coming to autopsy.^2^ That patient presented with asymmetrical rigidity at age 31 years and had a good motor response to dopaminergic therapy, but died at age 39 years. Pathologic study revealed nigral neuronal loss and gliosis, with a few LBs and Lewy neurites identified in the substantia nigra (SN), nucleus basalis of Meynert (NBM), and reticular formation of the brainstem, but not in the locus coeruleus (LC), amygdala, or hippocampus. This finding is interesting, because *PINK1* and *parkin* share a similar clinical phenotype, are believed to share a mechanistic pathway in the pathogenesis of PD,^3^ and, overwhelmingly, *parkin*-linked disease (PARK2) has been considered a non-LB disorder.

Case reports of genetically confirmed parkin disease and our recent case series of 5 unrelated *parkin* compound heterozygotes describe the pathology of parkin disease as a severe nigropathy with LBs present in only a minority of patients.^4^–^15^ The LC is consistently affected by neuronal loss, and tau pathology is occasionally described but is not a significant feature. Miyakawa and colleagues describe the first homozygous parkin disease case with definite LB pathology at postmortem examination.^16^ The female patient developed gait difficulty at age 61 years, and examination revealed all the cardinal features of PD. She appeared to respond well to dopaminergic therapy and developed motor fluctuations after 7 years, which were managed with bilateral deep-brain stimulation of the subthalamic nuclei. Her parents were first cousins, and her mother also possibly was affected. She was homozygous for deletions of exons 2 through 4 of *parkin*. Neuropathologic examination after 11 years of disease revealed a depigmented SN and LC. Histopathologic study confirmed SN neuronal loss with LBs and Lewy neurites in various brainstem nuclei (SN, LC, dorsal motor nucleus of the vagus, and NBM), the amygdaloid nucleus, the striatum, and the anterior cingulate cortex without evidence of other significant pathologies. Those authors suggested that the presence of truncated parkin protein (assumed to result from the homozygous deletion) may have resulted in LB formation; and they also considered the older age of onset but eventually concluded that coexistent sporadic PD may have led to the pathologic changes described.

There are several possible theories regarding the finding of LB pathology in both *parkin* and *PINK1*-related disease. First, it is possible that the LBs identified in these patents represent incidental LB disease (iLBD), which is described on postmortem examination in controls of increasing age,^17^–^19^ ie, the LBs represent presymptomatic PD, but the *parkin* or *PINK1* mutations have produced the clinical syndrome. The patient reported by Miyakawa et al., although aged > 70 years at death, had Braak stage IV^20^ LB pathology, which extends beyond what is considered iLBD. That brings us to the authors' conclusion that their case had coincidental sporadic PD. The patient's age at onset (61 years) was typical for sporadic PD, and it is possible the parkin phenotype had not yet become clinically established; we note that there exist occasional reports of parkin cases that did not manifest any signs of parkinsonism at ages well above that expected (>55 years),^8^,^21^ but those cases were compound heterozygous, and the possibility of reduced penetrance of 1 mutant allele would not apply in the homozygous situation. Conversely, the family history suggests that PD was not sporadic: the patient's parents were first cousins, and her mother possibly also suffered from parkinsonism. Like the cases reported by Farrer et al.^8^ and Pramstaller et al.,^12^ this produces an atypical inheritance pattern for parkin disease and makes us wonder about digenic disease and whether other genes, such as LRRK2, had been considered.

Age at disease onset is an intriguing explanation for the finding of LBs at postmortem examination in patients with parkin disease and is supported by the fact that no juvenile-onset cases have ever been reported with LBs postmortem.^22^,^23^ We have analyzed the age of onset in all reported cases and observed that the patients with LBs were significantly older when their symptoms began (mean age of onset: without LBs postmortem, 27 years; with LBs postmortem, 46 years).^15^ We can speculate that patients with a younger age at onset may have a different mechanism for dealing with abnormal protein accumulation; as a result, only neuronal dropout and gliosis are the neuropathologic findings when the disease overwhelms.

Before this case and that reported by Sasaki et al.^11^ it was assumed that large homozygous deletions of *parkin* were not associated with α-synuclein pathology because of the complete loss of parkin protein function, whereas incomplete loss of function is recognized with some missense mutations.^24^ The argument of partial protein function resulting from compound heterozygous mutations may explain the LBs found postmortem in the young onset PINK1 case^2^ but holds less well for this recent parkin case, in which homozygous deletions of exons 2 through 4 are likely to have rendered the resulting protein completely lacking in function.

Another interesting point is the patient's cardiac iodine-131 metaiodobenzylguanidine (MIBG) imaging. Decreased uptake of MIBG indicates cardiac sympathetic denervation and is typical for sporadic PD. The imaging was normal in this patient, as has been reported before in parkin-linked disease,^14^,^25^ in which it was suggested to signify the absence of LB pathology in parkinsonism cases.^14^ The relative preservation of epicardial sympathetic fascicles observed postmortem was in keeping with the antemortem imaging, but α-synuclein aggregates and LBs also were discovered in these structures.

The consistent neuropathologic findings in parkin disease remain those of severe SN and moderate LC neuronal loss. The variable findings of LBs and tau inclusions are not pathognomic or imperative for the condition. The discrepancy between areas of neuronal loss and areas of LB pathology described in the cardiac sympathetic system in this case provides additional support that these processes are not closely linked in parkin disease. The selective localization of the lesion in parkin disease is reminiscent of the localization of the lesion in 1-methyl-4-phenyl-1,2,3,6-tetrahydropyridine (MPTP)-induced parkinsonism.^26^ Parkin is a protein with the function of mitochondrial quality control,^3^ and MPTP is a mitochondrial toxin.^27^ The extent to which the nigral selectivity in MPTP-related disease reflects this mitochondrial selectivity or reflects selective uptake of the MPTP metabolite 1-methyl-4-phenylpyridinium (MPP+) into dopaminergic neurons^28^ is deserving of further study.

In summary, the clinical features and corresponding pathologic lesion of parkin disease appear to be distinct from those of sporadic PD, but the correlation remains unclear between the clinical syndrome of parkinsonism, the pathologic finding of LBs, and the underlying genetic predispositions of these features in the context of variable ages of onset.
